# Switching strategies with CGRP monoclonal antibodies: an observational study in a headache clinic

**DOI:** 10.3389/fneur.2026.1799960

**Published:** 2026-03-16

**Authors:** Marcos Polanco, Vicente González-Quintanilla, Jorge Madera, Sara Pérez-Pereda, Gabriel Gárate, Julio Pascual

**Affiliations:** Service of Neurology, University Hospital Marqués de Valdecilla, Universidad de Cantabria and IDIVAL, Santander, Spain

**Keywords:** anti-CGRP monoclonal antibodies, CGRP, migraine, preventive treatment, switch

## Abstract

**Background:**

Migraine is a disabling neurological disorder that greatly impacts quality of life and productivity. Calcitonin gene-related peptide (CGRP) monoclonal antibodies (mAbs) have recently improved preventive migraine therapy. However, some patients show limited efficacy, loss of response, or adverse effects, prompting interest in switching between mAbs.

**Objective:**

The objective of this work is to evaluate the effectiveness of switching between mAbs in chronic migraine/high-frequency episodic migraine (CM/HFEM) patients and to identify potential clinical predictors of response.

**Methods:**

This single-centre, real-life, prospective study was conducted in a tertiary hospital headache unit which includes patients with CM or HFEM who switched from one mAb to another without a treatment gap greater than 1 month. Monthly headache days (MHDs), monthly migraine days (MMDs), monthly analgesic consumption (MAC), and medication overuse (MO) were recorded 3 months before and at three and 6 months after switching. Subanalyses were performed based on the mechanism of action (anti-ligand vs. anti-receptor), reason for switch (lack or loss of efficacy), and clinical predictors.

**Results:**

Eighty-five patients (52.2 ± 9.6 years; 87.1% female; 95.3% CM) were included. At 3 months post-switch, reductions were observed in MHDs (−5.8 days, *p* < 0.001), MMDs (−5.8 days, *p* < 0.001), MAC (−5.6 days, *p* < 0.001), and MO (−22.2%, *p* = 0.001). At 6 months, these improvements persisted. A ≥ 50% response was achieved in 29.4% (25/85) of patients at month 3 and 32.9% (28/85) at month 6. One-third of patients whose switch included a change in mechanism of action responded, while none of those who underwent anti-ligand to anti-ligand switch showed a reduction of MHDs >50%. Both patients with loss and lack of efficacy to the first mAb improved significantly, though early response was more frequent among those with prior loss of efficacy. Obesity and concomitant tension-type headache were associated with poorer early response, while migraine with aura predicted better late response.

**Conclusion:**

Switching between mAbs could be an effective strategy for some CM and HFEM patients who fail initial mAb therapy, rescuing approximately one-third of non-responders. The therapeutic benefit seems to be greater when the second antibody targets a different mechanism of action. These real-world findings support the use of individualized switching strategies in refractory migraine management.

## 1 Introduction

Migraine is a disabling condition that affects approximately 15% of the population and has a remarkable impact on life quality and productivity (1–3). This impact is particularly high in patients with chronic migraine (CM) or with high frequency episodic migraine (HFEM) (1–3). Preventive strategies play a crucial role in reducing the frequency and severity of migraine attacks. Until a few years ago, prophylactic treatment of migraine was based on the use of different oral drugs including beta-blockers, antiepileptics and antidepressants and botulinum toxin type A in those patients who meet CM criteria (4). These therapies, though effective in some patients, are not exempt from adverse effects. The discovery of the key role of calcitonin gene-related peptide (CGRP) in the pathophysiology of migraine pain has led to the recent emergence of CGRP antagonists, which have clearly improved the management of this disease (4–6).

There are three monoclonal antibodies (mAbs) targeting the CGRP (galcanezumab, fremanezumab and eptinezumab) and one targeting its receptor (erenumab). The use of these specific therapies has led to a significant improvement in the quality of life of patients with migraine (4–6). However, there are still some situations where preventive treatment is not effective, loses effectiveness over time, or causes side effects that require its discontinuation. This has raised the question of whether a switch between monoclonal antibodies could be beneficial for these patients. The effectiveness of this therapeutic strategy has been explored in some real-world studies, showing positive results in a variable percentage of patients (7–17), but the underlying mechanism on which this potential benefit is based remains unknown.

Our aim was to contribute to the clarification of whether a switch between mAbs can be beneficial and to try to identify in which patient profile could a better switch response be expected.

## 2 Materials and methods

This is a single-centre, real-life study conducted in a headache unit of a tertiary hospital. Institutional review board approval (28/2020, 11 December) and written informed consent were obtained from every subject. Patients diagnosed with CM or HFEM according to current criteria (18) who started a mAb and switched to another without breaks of more than a month between both antibodies were included. The data collected includes records from early 2020 through the end of 2024.

In order to assess the response to mAb treatment after the switch, monthly headache days (MHDs), monthly migraine days (MMDs), monthly analgesic consumption (MAC), monthly non-steroidal anti-inflammatory drugs (NSAIDs), monthly triptans and number of concomitant preventive treatments were prospectively recorded in a calendar in medical records, as well as the existence of a concomitant criteria for medication overuse (18), in the 3 months prior switching and in the three and 6 months after the switch for all of the subjects. A subject was considered as responder when a reduction of at least 50% of MMDs or MHDs was observed after the switch and as super-responder when the reduction of MMDs or MHDs was higher than 75%. At the time of this analysis (June 2024), three monoclonal antibodies were available at our centre: erenumab, galzanezumab and fremanezumab. A structured review of electronic medical records of the included subjects was made by a neurologist with experience in headache to collect data related to demographic and clinical variables. Diagnosis of anxiety-depression was taken from the medical records and had to be performed by a psychiatrist.

Secondly, considering the different mechanisms of action of the available mAbs, 3 types of switches were examined: from anti-ligand to anti-ligand, from anti-receptor to anti-ligand and viceversa. To determine the most beneficial switch therapeutic strategy we performed a comparative analysis of the main variables of the study (MMDs, MHDs, MAC and presence of MO) among the three subgroups. Also, a comparative analysis of the switch response was performed between those subjects in whom the switch was motivated by a loss of effectiveness of the first mAb (understood as a significant increase in the MHDs and/or MMDs or sustained perception of loss of effectiveness by the patient, after having achieved a response of at least 25% reduction in MHDs and/or MMDs during the first 6 months of treatment) and those who did not respond to the first antiCGRP drug (absence of at least 25% reduction in their MMDs or MHDs in the first 6 months of treatment).

Finally, to try to identify possible clinical or demographic characteristics that could determine response, we performed a comparative between those who achieved at least a 50% response with the switch among mAbs and those who did not.

### 2.1 Statistical analyses

No *a priori* sample size calculation was performed, as this was an observational study that included all consecutive patients meeting the inclusion criteria at our centre during the study period. All statistical analyses were pre-planned and constitute the primary analysis of the study; no post-hoc exploratory analyses were carried out.

A descriptive analysis was performed in which the qualitative variables are presented with their absolute (n) and relative (percentage, %) frequencies, and the quantitative variables are described by a central tendency measure (mean or median) and a dispersion measure (standard deviation or interquartile range), according to their normal or non-normal distribution, respectively. In the presentation of results, data dispersion will be expressed using the ± symbol to denote the standard deviation. The distribution of continuous quantitative variables was analyzed using the Kolmogorov–Smirnov test.

For comparative analysis, *t*-test was used to compare continuous variables with normal distribution from both independent and paired samples. In the case of ordinal variables or continuous variables with non-normal distribution, Wilcoxon test was used when the samples were paired and Mann–Whitney *U* test when they were independent. Finally, Chi-square or Fisher’s exact test (for small, expected cell counts) was used to compare independent categorical variables, while McNemar test was used if the categorical variables came from paired samples. All tests were two-tailed and statistical significance was set at *p* < 0.05. Data analysis was performed using SPSS version 25 (IBM Corp., Armonk, NY, USA).

## 3 Results

At the time of this analysis, 472 patients with refractory HFEM or CM had been treated with at least one mAb in our Headache Unit, of whom 173 (36.7%) had received at least two anti-CGRP therapies. After reviewing the clinical charts of these subjects, 88 patients were excluded due to treatment gaps of more than 1 month during the switch period.

**Figure 1:**
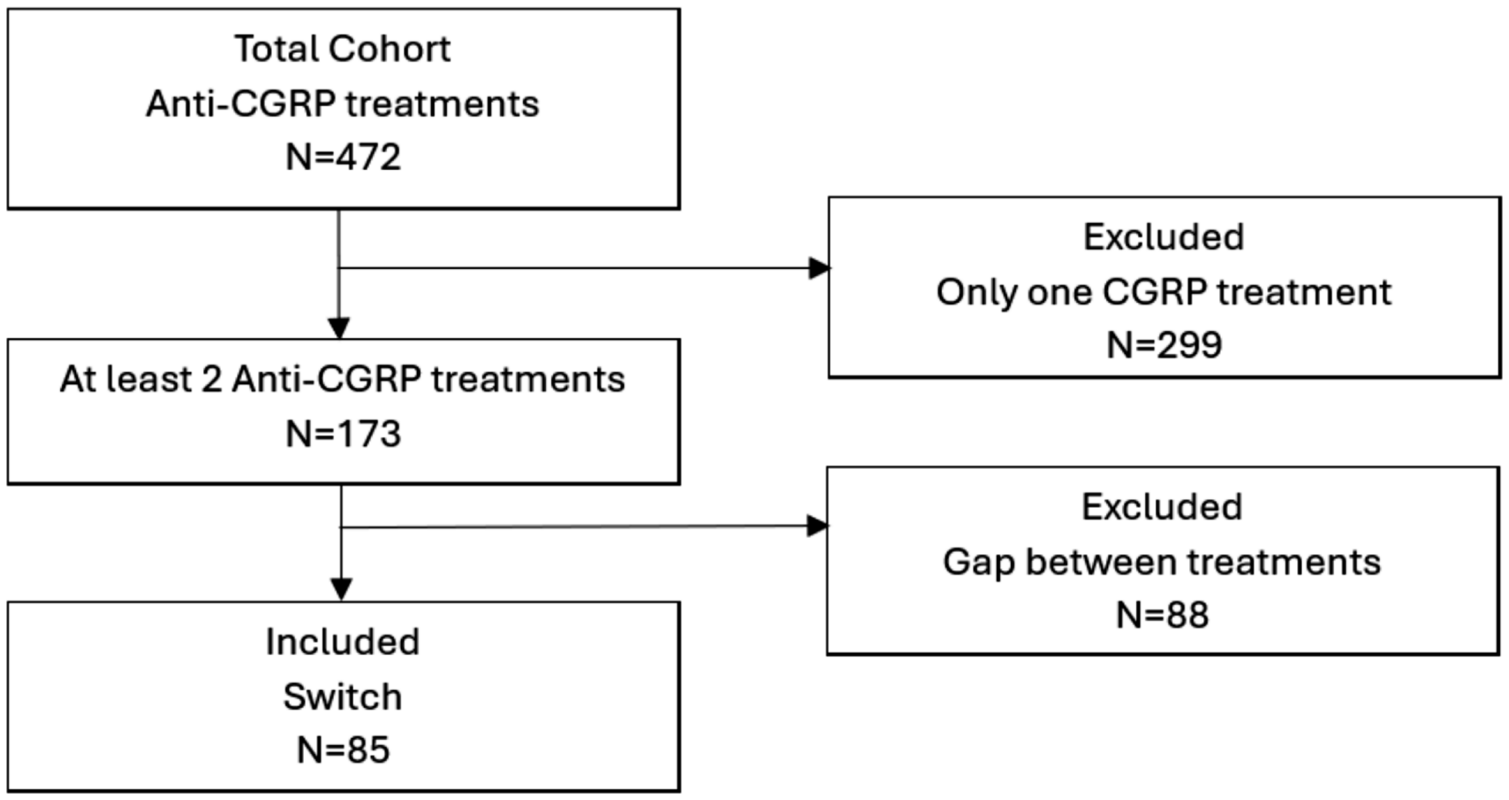
Flowchart of the study. Patients with gaps of the switch between mAbs longer than 1 month were excluded.

Therefore, 85 patients were finally included in the present study (Figure 1).

### 3.1 Baseline characteristics

Mean age of the sample was 52.2 ± 9.6 years old and 74 (87.1%) were female. Out of the 85 patients, 81 (95.3%) were diagnosed with CM and the remaining 4 (4.7%) with HFEM, with a mean duration of their condition of 31.3 ± 12.4 years. The mean number of years in the situation of HFEM or CM was 12.8 ± 10.4 years. The most frequent comorbidities associated with these subjects were anxiety or depression-anxiety (50, 58.8%), obesity (16, 18.8%) and fibromyalgia (16, 18.8%) (Table 1). Thirty-four (45.9%) of the 74 women had menopause.

**Table 1:** Baseline characteristics of the study sample.

Demographics		
Age	52.2 ± 9.6	
Sex	Females	74 (87.1%)
	Males	11 (12.9%)
Main comorbidities		
Anxiety-depressive syndrome	50 (58.8%)	
Hypertension	19 (22.4%)	
Obesity	16 (18.8%)	
Fibromyalgia	16 (18.8%)	
Smoking	12 (15.6%)	
Migraine characteristics		
Diagnosis	Chronic migraine	81 (95.3%)
	High frequency migraine	4 (4.7%)
Age of onset	19.72 ± 10.75	
Years with migraine	31.3 ± 12.4	
Years of HFEM or CM	12.8 ± 10.4	
Concomitant tension-type headache	28 (32.9%)	
Aura	28 (32.9%)	
Photophobia	23 (28.4%)	
Allodynia	23 (29.1%)	
Localization	Unilateral	29 (34.5%)
	Bilateral	1 (1.2%)
	Uni and bilateral	53 (63.1%)

Regarding previous use of oral preventive treatments, the most used were amitriptyline (78, 91.8%), topiramate (76, 89.4%) and beta-blockers (75, 88.2%). A total of 74 (87.1%) patients had also received onabotulinumtoxin A. As first mAb, the most frequently used was erenumab (51, 60%) followed by galcanezumab (30, 35.3%) and fremanezumab (4, 4.7%). Baseline characteristics of the total sample are shown in Table 1.

### 3.2 Global switch analysis

Median exposure to the first mAb was 12 months (IQR 6–21.5). At switch time the mean MHDs of the sample was 22.5 ± 8.1 days, that of MMDs 18.8 ± 8.5 days, and for MAC was 19.4 ± 8.6 days. A total of 46 patients (54.1%) met criteria for medication overuse (MO). The mean monthly intake of triptans was 13.8 ± 8.2 days and 22.4 ± 7.9 days for NSAIDs. Prior to antiCGRP antibody switching, 56 patients (66.7%) were also receiving preventive treatment with oral drugs or onabotulinumtoxin A. At month 3 post-switch a significant reduction of the mean MHDs (−5.8 days; *p* < 0.000), MMDs (−5.84 days; *p* < 0.001), MAC (−5.55 days; *p* < 0.001) and number of patients with MO (−22.2%; *p* = 0.001) was observed. Of the total sample, 29.4% were considered responders and 13.2% super-responders, while 41.2% experienced a reduction of at least 25% of their MHDs and/or MMDs. The improvement remained 6 months after the switch, showing similar or slightly enhanced results (Table 2). There was also a significant decrease in the monthly intake of NSAIDs at both the third (−8.2 days; *p* = 0.002) and sixth month (−7.2 days; *p* = 0.002) after the switch, although the numerical decrease in monthly triptan intake was not significant (13.8 vs. 12.21; *p* = 0.165 and 13.8 vs. 9.46; *p* = 0.057). In addition, no significant reduction in the number of subjects who associated a concomitant use of other preventive drugs was observed (Table 2).

**Table 2:** Comparative analysis of the switch response in the total sample.

Variables	Month 0	Month 3	*p*	Month 6	*p*
MHDs	22.5 ± 8.1	16.7 ± 10.1	*<0.001* ^b,*^	16.8 ± 10.4	*<0.001* ^a,*^
MMDs	18.8 ± 8.5	13 ± 9.2	*<0.001* ^a,*^	11.7 ± 9.3	*<0.001* ^a,*^
MAC	19.4 ± 8.6	14.42 ± 9.7	*<0.001* ^b,*^	13.6 ± 9.9	*<0.001* ^a,*^
MO	46 (54.1%)	24/68 (35.3%)	*0.001* ^c,*^	18/65(27.7%)	*<0.001* ^c,*^
Triptans	13.8 ± 8.2	12.21 ± 10.4	0.165*^b^*	9.46 ± 7.8	0.057^b,*^
NSAIDs	22.39 ± 7.9	14.21 ± 10.6	*0.002* ^a,*^	15.18 ± 9.4	*0.002* ^a,*^
Concomitant preventive drugs	56 (66.7%)	47/68 (69.1%)	*p* > 0.999^c^	50/65 (76.9%)	0.453^c^
Botulinum toxin type A	18 (21.2%)	14/68 (20.6%)	*p* > 0.999^c^	14/65 (21.9%)	*p* > 0.999^c^

^a^Paired *t*-test, ^b^Wilcoxon test, ^c^McNemar test. *Statistically significant result.

MHDs, Monthly Headache Days; MMDs, Monthly Migraine Days; MAC, Monthly Analgesic Comsuption; MO, Medication Overuse.

During the follow-up period after switch, mAb therapy was discontinued in 12 (14.1%) subjects due to lack of effectiveness and in only 1 (1.2%) because of adverse events (allergic reaction). AntiCGRP therapy was withdrawn in further 13 patients (21.0%) due to lack of effectiveness during the next 3 following months (months 4–6). The most frequent adverse event reported during this period was constipation (10.9%).

### 3.3 Comparative subanalyses

#### 3.3.1 According to the mechanism of action

Of the whole sample, 51 (60.0%) subjects underwent from an anti-receptor to an anti-ligand mAb, 24 (28.2%) from an anti-ligand to an anti-receptor antiCGRP and only 10 (11.8%) from an anti-ligand to another anti-ligand mAb. Switch was motivated by lack of efficacy in 79 (92.9%) patients and by intolerance/adverse events in 6 (7.1%). Prior to switch, the number of MHDs, MMDs, MAC and concomitant MO did not differ significantly between subgroups (*p* > 0.05).

**Figure 2:**
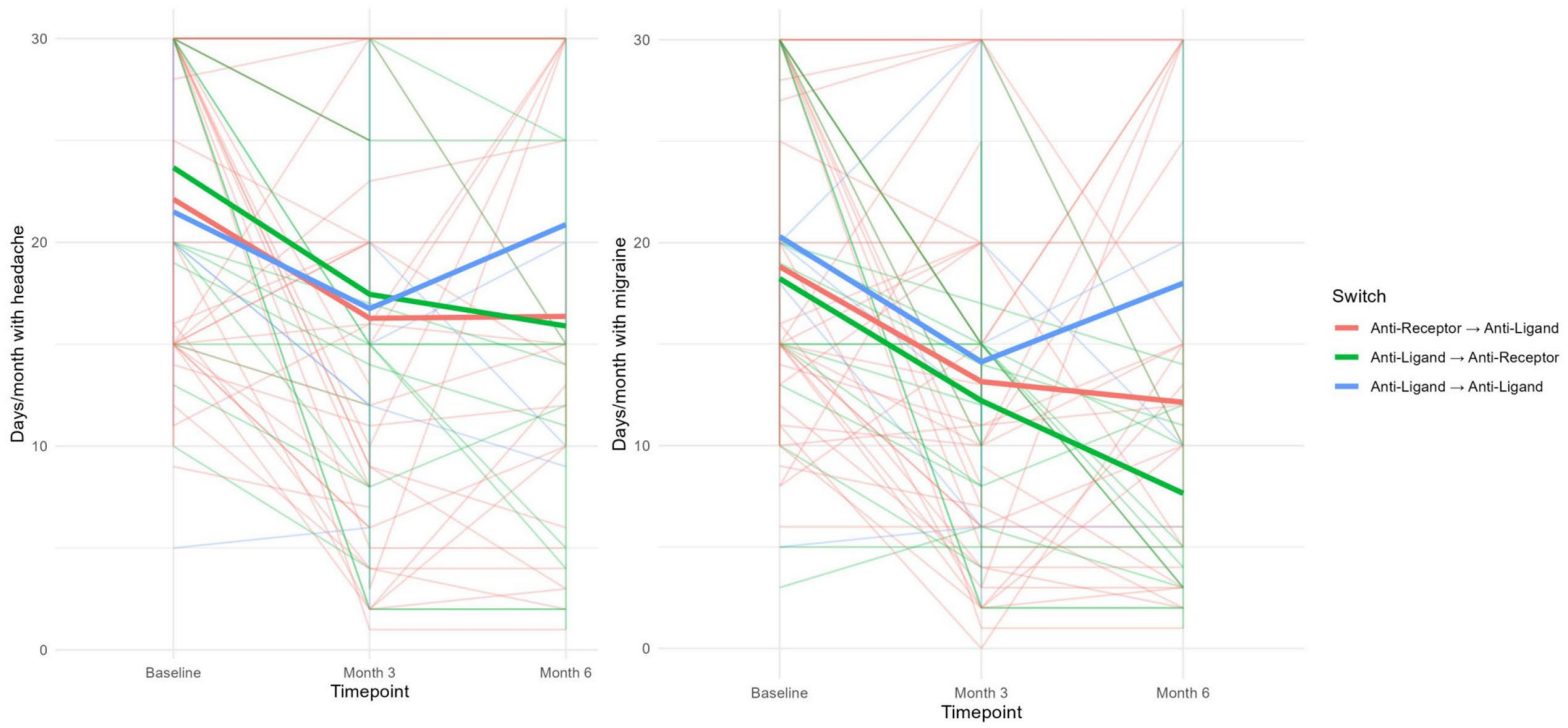
Average MMDs (right) and MHDs (left) before and 3 and 6 months after switching, according to the underlying mechanism of action.

Three months after the switch, a significant reduction in MMDs and MHDs was observed in each of the groups, regardless of the type of switch performed. However, this significant response was only maintained at month 6 in those subjects who had received a second mAb with a different mechanism of action (Figure 2).

Similarly, only those subjects who received a second mAb with a different mechanism of action experienced a significant reduction in MAC, both at third and sixth month post-switch, and in concomitant MO at sixth month post-switch (Table 3).

**Table 3:** Comparative analysis of the switch response based on the underlying mechanism of action.

Variables	M0	M3	*p*	M6	*p*
Anti-receptor to anti-ligand mAb (*N* = 51)					
MHDs	22.2 ± 8	16.3 ± 10.7	**0.003** ^b,*^	16.4 ± 10.8	**0.002** ^b,*^
MMDs	18.8 ± 8.5	13.2 ± 10.2	**0.001** ^b,*^	12.1 ± 9.6	**<0.001** ^b,*^
MAC	19.3 ± 8.6	14.3 ± 10.4	**0.012** ^b,*^	13.5 ± 10.14	**0.002** ^b,*^
MO	27 (52.94%)	14/38 (36.8%)	**0.039** ^c,*^	10/36 (36%)	**0.006** ^c,*^
Anti-ligand to anti-receptor (*N* = 24)					
MHDs	23.7 ± 8.3	17.5 ± 9.5	**0.001** ^b,*^	15.9 ± 10.4	**0.004** ^b,*^
MMDs	18.2 ± 9.4	12.2 ± 7.3	**0.002** ^b,*^	7.7 ± 6.9	**0.002** ^b,*^
MAC	20.9 ± 8.5	14.4 ± 8.4	**0.001** ^b,*^	11.4 ± 9.3	**0.012** ^b,*^
MO	13 (54.2%)	6 (30%)	**0.031** ^c,*^	4 (21.1%)	0.125^c^
Anti-ligand to anti-ligand (*N* = 10)					
MHDs	21.5 ± 8.7	16.8 ± 9.2	0.08^b^	20.9 ± 8.3	0.500^b^
MMDs	20.3 ± 8.2	14.13 ± 8.1	0.176^b^	18 ± 8.8	0.043^b^
MAC	21.4 ± 9.3	15.8 ± 9.7	0.180^b^	20.8 ± 8.1	0.109^b^
MO	6 (60.0%)	4/8 (50.0%)	*p* > 0.999^c^	4/8 (50.0%)	*p* > 0.999 *^c^*

^b^Wilcoxon test, ^c^McNemar test. The asterisk and bold type interchangeably to highlight statistically significant results.

MHDs, Monthly Headache Days; MMDs, Monthly Migraine Days; MAC, Monthly Analgesic Comsuption; MO, Medication Overuse.

Regarding response rate, 35% of subjects who underwent an anti-receptor to anti-ligand switch showed a 50% reduction in their MHDs, while the reverse switch showed a 50% response rate in 30%. None of the patients who underwent anti-ligand to anti-ligand switch experienced a reduction in their MHDs equal to or greater than 50%. Results obtained from the comparative analysis in each of the subgroups are shown in Table 3.

#### 3.3.2 According to previous mAb response

Switch response was also evaluated between those subjects who had no response to the first mAb from the beginning and those who had experienced a loss of effectiveness of the first treatment. Both subgroups had comparable baseline characteristics, MHDs, MMDs, MAC and MO rates before switching.

Either lack and loss of effectiveness subgroups presented a significant reduction in MMD and MHD months 3 and 6. Regarding MAC, subjects who had experienced a loss of effectiveness to the first mAb showed a significant reduction of this endpoint at month 3 maintained over the next 3 months, whereas those who had not responded to the first mAb from the beginning only showed a significant reduction of MAC at month 6 (Table 4).

**Table 4:** Comparative analysis of the response to the switch based on the kind of response to the first mAB.

Variables	Lack of effectiveness from the beginning (*N* = 32)					Loss of effectiveness after initial response (*N* = 53)				
	M0	M3	*p*	M6	*p*	M0	M3	*p*	M6	*p*
MHDs	22.8 ± 8.6	19.1 ± 0.6	**0.022** ^b,*^	16.7 ± 11.0	**0.004** ^b,*^	22.3 ± 7.8	15.3 ± 9.6	**<0.001** ^b,*^	16.7 ± 10.2	**0.001** ^b,*^
MMDs	18.7 ± 9.3	13.4 ± 9.2	**0.008** ^b,*^	12.2 ± 10.2	**0.002** ^b,*^	18.9 ± 8.2	12.8 ± 9.2	**<0.001** ^b,*^	11.4 ± 8.9	**<0.001** ^b,*^
MAC	19.5 ± 8.9	16.6 ± 10.5	0.148^b^	15.8 ± 11.4	**0.041** ^b,*^	20.3 ± 8.4	13.1 ± 10.9	**<0.001** ^b,*^	12.3 ± 8.8	**<0.001** ^b,*^
MOH	17 (58.6%)	11/25 (44.0%)	0.500^c^	8/25 (32.0%)	0.219^c^	29 (56.9%)	13/43 (30.2%)	0.003^c^	10/40 (25.0%)	0.002^c^

MHDs, Monthly Headache Days; MMDs, Monthly Migraine Days; MAC, Monthly Analgesic Comsuption; MO, Medication Overuse.

^b^Wilcoxon test, ^c^McNemar test. The asterisk and bold type interchangeably to highlight statistically significant results.

Finally, 20% of patients who failed their initial monoclonal antibody treatment achieved a 50% response rate at 3 months post-switch, rising to 32% at 6 months. Among patients who experienced loss of response to initial treatment, 34.9% achieved a 50% response rate at month 3, with 32.5% maintaining this response at month 6. Response rates at both time points showed no significant difference between the two subgroups.

#### 3.3.3 According to switch response

To determine possible baseline factors conditioning switch response, we performed a comparative analysis of baseline characteristics between subjects who were considered responders (decrease of at least 50% of their MMDs or MHDs pre-switch) and those who did not respond, at 3 months (early response) and 6 months (late response) of follow-up.

Subjects with obesity had a significantly lower early response to switch than those with a BMI < 25 (0% vs. 28.6%, *p* < 0.001), although the percentage of obese subjects with late response did not significantly differ compared to late responders without obesity (33.3% vs. 35.7%). Even though the difference was not significant, those subjects with another concomitant tension-type headache responded less frequently in an early manner (11.1% vs. 34.7%, *p* = 0.058). On the other hand, and in the absence of an early response, a late response to switch was more frequent in those subjects who presented migraine with aura (83.3% vs. 14.3%, *p* = 0.002) and in those with a later migraine onset (22.3 vs. 16.2 years old, *p* = 0.044). No significant differences were observed with respect to the rest of the characteristics studied.

## 4 Discussion

Patients’ responses to preventive therapies significantly influence the choice of alternative switch therapies in migraine treatment. When patients do not respond adequately to a first mAb, switching to another is a common approach in clinical practice, especially in desperate patients with refractory CM, but the effectiveness of this strategy has not been tested in controlled trials. This real-world study is consistent with and complements previous experiences suggesting that a switch between mAbs may be beneficial in some patients with failure or loss of effectiveness to the first mAb. In our experience, almost one-third of patients responded to the mAb switch (50% reduction) at months 3 and 6. Our results show that the change of mAb led to a significant reduction of up to 5.8 days of headache and migraine at month 3 and this reduction was maintained at month 6. Consequently, the number of days of symptomatic medication was also significantly reduced (−5.5 days), and the proportion of patients meeting MO criteria was also significantly reduced at months 3 and 6 of the change between mAbs.

A few studies have pointed out that switching between mAbs can lead to a reduction in MHDs at months 3 and 6 with variable ranges (7–17). Reduction in MHDs in such studies has ranged from −1.9 to −6 days at month 3 and to −7 at month 6. These findings are similar or slightly lower than those shown in our study (−5.8 MHDs).

Regarding the reduction of at least 50% in MHDs at month 3, this was observed on average in 18.0% (13.6–33.0%) of patients (7, 9–11, 13–15), which is in line with our findings, where 50% response at month 3 was observed in 29.4% of patients. The 50% reduction at month 6 of treatment has been less studied, and published findings reflect a reduction of 15.4%, which is lower than our results (7). Most studies only evaluate the response to mAb switching at month 3 and do not perform medium-term follow-up at month 6, which means that the number of responders may be lower as those who respond late are not included. Furthermore, not all studies have three monoclonal antibodies with three different types of switch or have excluded patients whose switch was not sequential (7–17).

The immediate explanation that justifies the response seen in some patients with the switch could be a change in mAb target of action and this was specifically analyzed in this series. In fact, our largest group is the one that switched from a receptor to ligand blocking mAb because erenumab was the first anti-CGRP antibody approved at our hospital. Both anti-receptor to anti-ligand and anti-ligand to anti-receptor switching showed a significant reduction in MHDs MMDs, MAC and NSAID consumption at 3 months. This improvement was largely maintained at 6 months, except for the reduction in concomitant MO in the anti-ligand to anti-receptor switch group. In contrast, subjects who switched from one anti-ligand to another anti-ligand mAb showed a non-significant reduction in MHDs, MMDs, MAC and NSAID consumption. This suggests that some patients respond to a specific mechanism of action and not to another. Therefore, in those who have failed one mechanism of action, it may not be worthwhile to attempt a second treatment with another mAb of the same mechanism. Instead, our data indicate that switching to a different mechanism of action would be more beneficial.

A second factor, untested in previous studies, which could influence the result of the switch, is the potential difference in its effectiveness in patients with lack or loss of effectiveness. The patient and clinician perception are different in switching due to loss of effectiveness after an initial response or to lack of effectiveness of mAbs from the start, and the decision to switch due to lack of effectiveness can be based on either criteria. These two scenarios may have different considerations and strategies in the decision of switching therapies and perhaps different response rates. Interestingly, in our population, both subgroups experienced a significant reduction in MMDs, MHDs, MAC and NSAID consumption at both 3 and 6 months after switching. This sustained improvement suggests that switching from one mAb to another is a viable strategy for some patients, regardless of whether their initial problem was a lack of response from the beginning or a response that diminished over time. In terms of magnitude, although the reduction was significant in both groups, patients with loss of effectiveness to the first antibody experienced a greater reduction in MHDs than those with lack of effectiveness (−7 vs. − 3.7) at month 3. These differences were equalized at month 6 of treatment. Similarly, patients with progressive loss of effectiveness responded better at month 3 (≥50% response rate: 20 vs. 34.9%), but not at month 6 (32% vs. 32.5%). This could be justified because some patients with loss of effectiveness to the first monoclonal antibody also experience the same efficiency loss phenomenon to the second monoclonal antibody; in fact, MHDs increased by +1.4 days between month 3 and 6 in this group.

More studies are needed to know why some patients initially respond well to an anti-CGRP mAb but experience a loss of effectiveness over time. This can be due to various factors, including changes in the patient’s condition or the development of tolerance to the medication (16). Our results indicate that, even in these cases, switching to another mAb may restore the therapeutic effect in around one-third of patients. The choice of the new mAb can depend on the specific target (ligand vs. receptor) and the patient’s previous response pattern.

Finally, we tried to identify factors associated with response to switch strategy. Our results indicate that certain clinical characteristics can influence the likelihood of a successful response to mAb switch. We observed that obesity was associated with a worse response to the change in treatment at month 3, although these differences evened out at month 6. This could be explained by the fact that patients with obesity have a greater volume of distribution for the drug; in addition, it is known that this type of patient has an inflammatory state *per se* that worsens migraine and response to treatment. Obesity has been associated with a poorer response to anti-CGRP therapies and may need to be considered when deciding to switch therapies (19, 20). These results also raise the possibility that obese CM or HFEM patients could need higher doses of CGRP antagonists to obtain effectiveness. The presence of concomitant tension-type headache was another factor associated with poorer response to antibody switching. This could be justified by the fact that these patients could have less pure migraine and associate other types of headaches that may increase the baseline headache days and do not respond to antibodies specifically directed against neuropeptides involved in the pathophysiology of migraine. This supports the idea that patients with aura, with no other associated factors, and consequently with purer migraine attack, had a better late response (month 6). Therefore, even if no response is evident in the first quarter, it seems reasonable maintaining the mAb up to 6 months, especially in those who show a purer migraine phenotype as exemplified by the aura. Unlike other works, we did not find a relationship with other parameters, such as the presence of unilateral pain combined with unilateral cranial autonomic symptoms or allodynia (20). Further research, with a higher number of cases, is needed to explore predictors related to switching between different anti-CGRP treatments.

The limitations of this study include the nature of this real-world or its single-centre design. Being and observational, uncontrolled study concomitant preventive treatments, a possible carry-over effect of the previous mAbs, regression to the mean and placebo effects should be included among the potential confounders A larger sample size would be necessary to obtain more consistent results, especially in cases of antibody switch without a change in the mechanism of action, and findings in subgroups should be considered as exploratory. Among the strengths of the study, it is worth highlighting that we have complete and prospective data from a highly variable sample of patients and the study was conducted with three mAbs out of the four currently launched. These observations can be applied to routine clinical practice as they are based on a real-world study. In conclusion, this study demonstrates that switching between mAb in patients who have failed initial CGRP treatment can rescue up to approximately 30% of patients.

This therapeutic switch can occur in either direction: from receptor-blocking to ligand-blocking mechanisms or viceversa. In definitive terms, the change in the mechanism of action appears to be more beneficial. Less consistently, ligand-to-ligand switching, without modifying the mechanism of action, may be beneficial but its confirmation requires a study with a bigger number of patients. These findings are based on our real-life experience and can therefore be applied to migraine patients undergoing treatment with mAbs in daily clinical headache practice. Furthermore, in contrast to clinical trials, it includes the most common comorbidities in these patients. In conclusion, the choice of the new mAb can depend on the specific target (ligand vs. receptor) and the patient’s previous response pattern (21).

In definitive terms, our findings suggest that switching the mechanism of action appears to provide greater therapeutic benefit compared to switching between agents with the same target. Less consistently, switching between anti-ligand mAbs without changing the mechanism of action may also provide clinical advantages, although confirmation requires validation studies with larger patient populations and a longer duration of therapy (21). These observations are derived from real-world clinical experience and can therefore be applied to migraine patients receiving mAb therapy in routine headache practice. Moreover, unlike randomized controlled clinical trials with strict inclusion criteria, our study includes patients with common medical and psychiatric comorbidities that are frequently encountered in routine practice, thereby enhancing the generalizability of our findings to broader patient populations.

In conclusion, sequential mAb therapy following initial treatment failure represents a clinically meaningful strategy, with approximately 30% of patients achieving response. Treatment selection should be individualized based on two key factors: the molecular target of the initial mAb (ligand versus receptor), with preference for switching mechanism of action when feasible, and the patient’s pattern of treatment failure (primary non-response versus secondary loss of efficacy).

## Data Availability

The raw data supporting the conclusions of this article will be made available by the authors, without undue reservation.
