# Correction: Switching strategies with CGRP monoclonal antibodies: an observational study in a headache clinic

**DOI:** 10.3389/fneur.2026.1845315

**Published:** 2026-04-16

**Authors:** Marcos Polanco, Vicente González-Quintanilla, Jorge Madera, Sara Pérez-Pereda, Gabriel Gárate, Julio Pascual

**Affiliations:** Service of Neurology, University Hospital Marqués de Valdecilla, Universidad de Cantabria and IDIVAL, Santander, Spain

**Keywords:** anti-CGRP monoclonal antibodies, CGRP, migraine, preventive treatment, switch

In the published article, there was an error in Figure 2: the graphs within the figure were duplicated. The corrected Figure 2 appears below:

**Figure 2:**
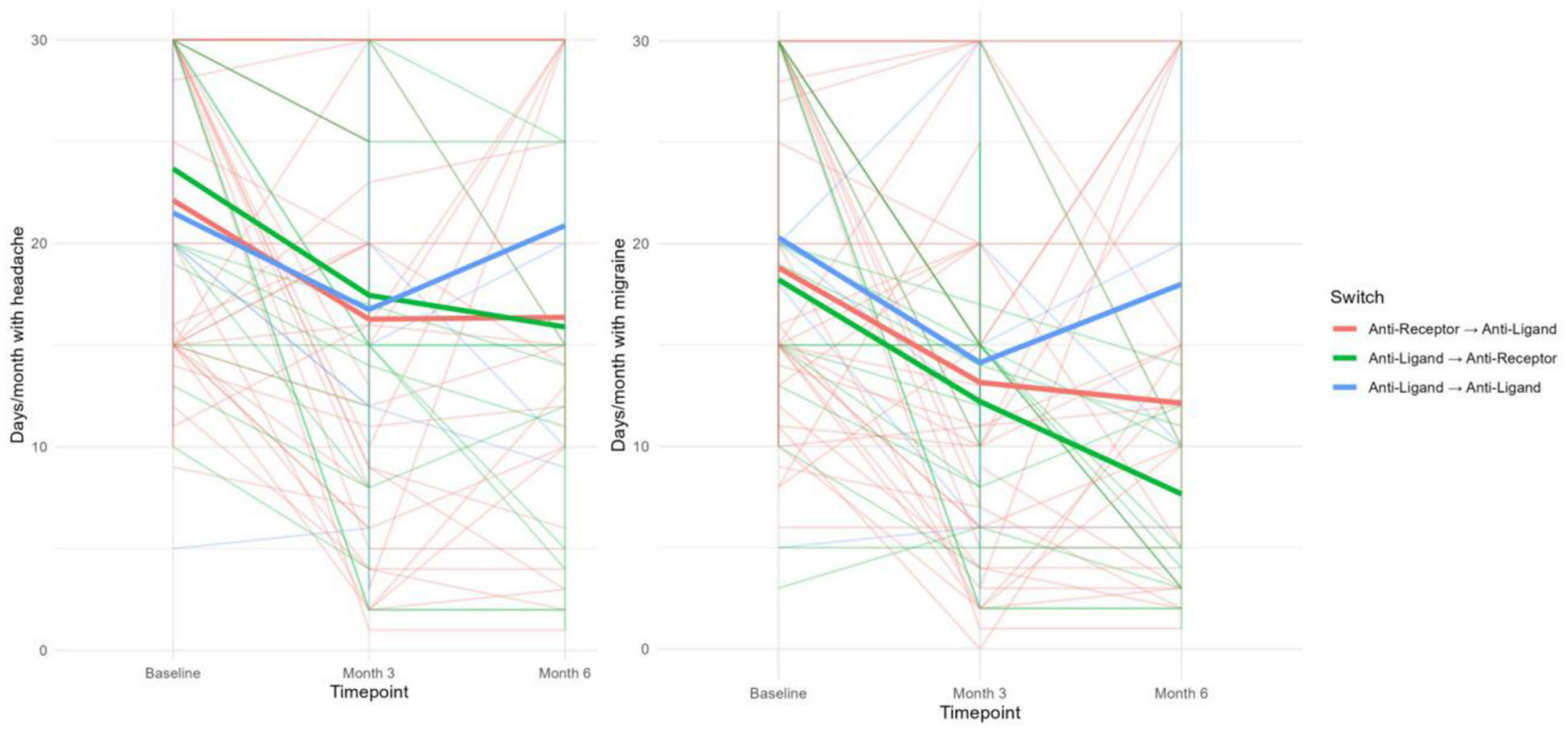
Average MMDs **(Right)** and MHDs **(Left)** before and 3 and 6 months after switching, according to the underlying mechanism of action.

The original version of this article has been updated.

